# NrCAM-deficient mice exposed to chronic stress exhibit disrupted latent inhibition, a hallmark of schizophrenia

**DOI:** 10.3389/fnbeh.2024.1373556

**Published:** 2024-03-27

**Authors:** Mona Buhusi, Colten K. Brown, Catalin V. Buhusi

**Affiliations:** Interdisciplinary Program in Neuroscience, Department of Psychology, Utah State University, Logan, UT, United States

**Keywords:** c-Fos, chronic stress, latent inhibition, neuronal cell adhesion molecule (NrCAM), orbitofrontal cortex, infralimbic cortex, nucleus accumbens, schizophrenia

## Abstract

The neuronal cell adhesion molecule (NrCAM) is widely expressed and has important physiological functions in the nervous system across the lifespan, from axonal growth and guidance to spine and synaptic pruning, to organization of proteins at the nodes of Ranvier. NrCAM lies at the core of a functional protein network where multiple targets (including NrCAM itself) have been associated with schizophrenia. Here we investigated the effects of chronic unpredictable stress on latent inhibition, a measure of selective attention and learning which shows alterations in schizophrenia, in NrCAM knockout (KO) mice and their wild-type littermate controls (WT). Under baseline experimental conditions both NrCAM KO and WT mice expressed robust latent inhibition (*p* = 0.001). However, following chronic unpredictable stress, WT mice (*p* = 0.002), but not NrCAM KO mice (*F* < 1), expressed latent inhibition. Analyses of neuronal activation (c-Fos positive counts) in key brain regions relevant to latent inhibition indicated four types of effects: a single hit by genotype in IL cortex (*p* = 0.0001), a single hit by stress in Acb-shell (*p* = 0.031), a dual hit stress x genotype in mOFC (*p* = 0.008), vOFC (*p* = 0.020), and Acb-core (*p* = 0.032), and no effect in PrL cortex (*p* > 0.141). These results indicating a pattern of differential effects of genotype and stress support a complex stress × genotype interaction model and a role for NrCAM in stress-induced pathological behaviors relevant to schizophrenia and other psychiatric disorders.

## 1 Introduction

Schizophrenia (SZ) is a severe psychiatric disorder affecting approximately 1% of the population worldwide (Kahn et al., 2015). With an onset in early adulthood, and a chronic course with a diverse array of symptoms (delusions, hallucinations, disorganized speech and behavior, flat affect, decreased motivation, anhedonia, impairments in attention, working memory and executive functions) (American Psychiatric Association, 2013), SZ often leads to social and occupational dysfunction, and thus impacts not only the individual but the entire society (e.g., the excess economic burden of SZ in the US was estimated at $343 billion, Kadakia et al., 2022). Recent research aimed at elucidating the etiology of SZ has revealed a complex interplay of genetic and environmental factors (Birnbaum and Weinberger, 2017). The disorder is characterized by high heritability (over 80%) (Hilker et al., 2018), but the genetic architecture is heterogeneous, involving both rare damaging variants (inherited and *de novo*) that highly increase risk and numerous common variants with small to moderate effects (Birnbaum and Weinberger, 2017; Wahbeh and Avramopoulos, 2021). In addition to genetically-induced vulnerabilities, exposure to challenging environmental factors at various developmental stages (prenatal or perinatal life, adolescence, or adulthood) contribute to the emergence of SZ (Stilo and Murray, 2019; Richetto and Meyer, 2021).

Weinberger (1987) proposed a ‘neurodevelopmental model’ of SZ, suggesting that alterations in normal brain development lead to an altered brain developmental trajectory that is sensitive to factors associated with development and environmental experience, consequently leading to the emergence of schizophrenia in early adulthood. If the original neurodevelopmental model of SZ was based mostly on epidemiological evidence linking the disorder to prenatal and early postnatal life, recent analyses have revealed that many genes associated with SZ influence early neurodevelopmental processes such as neuronal migration, differentiation, maturation, and neuronal connectivity.

One of the genes associated with an increased risk of developing SZ is the Neuronal Cell Adhesion Molecule (NrCAM) gene (Kim et al., 2009; Ayalew et al., 2012; Karimian et al., 2020), a gene coding for a cell adhesion molecule widely expressed in the nervous system, and with important physiological functions from early development throughout the lifespan (Sakurai et al., 2001; Dai et al., 2013; Mohan et al., 2018, 2019a). NrCAM lies at the core of a functional protein network where multiple targets have been associated with SZ: First, NrCAM binds ankyrins, versatile adapters between membrane proteins and the cytoskeleton at the axon hillock and nodes of Ranvier, involved in neuronal excitability. Giant ankyrins promote GABAergic synapse stability (Tseng et al., 2015). Both the ANK2 and ANK3 genes show strong associations with SZ (Luoni et al., 2016; Stevens and Rasband, 2021). Second, NrCAM is also associated with SAP97 (Dirks et al., 2006), an adapter for glutamate receptors (Waites et al., 2009; Li et al., 2011); SAP97 is associated with SZ, in particular with neurocognitive dysfunctions (Uezato et al., 2017; Xu et al., 2018, 2020, 2021), and SZ-associated SAP97 mutations increase glutamatergic synapse strength in the dentate gyrus and impair contextual episodic memory (Kay et al., 2022). Third, NrCAM is expressed in cortical astrocytes and neurons and forms perisynaptic contacts at inhibitory synapses. Depletion of astrocytic NrCAM reduces the numbers and function of inhibitory synapses (Takano et al., 2020), providing a possible mechanism for the cortical excitation-inhibition imbalance thought to underlie some of the SZ phenotypes (Howes and Shatalina, 2022). Fourth, a recent study assessing pituitary stress-induced gene regulation reported changes in expression for NrCAM and NrCAM-interacting proteins (ANK3, PAK2) after social defeat stress in rodents (Olsen et al., 2022); the same study reports that a variant of the human NrCAM gene is associated with negative affect after abusive supervision (Olsen et al., 2022). Finally, NrCAM is decreased in serum from SZ patients (Schwarz et al., 2012). These findings support roles for NrCAM in both developmental vulnerability and altered responses to environmental stressors, making it an intriguing target for SZ research.

In order to investigate the neuropathological mechanisms and to develop effective new strategies to treat SZ, valid animal models are required which accurately model the disorder, and ideally provide construct, face and predictive validity. Given that NrCAM-deficient mice provide genetic construct validity, we have proceeded to analyze their face validity—expression of SZ phenotypes—using translational behavioral tasks. In the present study NrCAM KO mice were evaluated for expression of an attentional phenomenon whose deficit is considered a hallmark of SZ: *Latent Inhibition* (LI) (Lubow and Moore, 1959; Lubow, 1989; Lubow and Gewirtz, 1995). LI is defined as a decrement in associability of a stimulus (slower learning of a conditioned stimulus (CS) – unconditioned stimulus (US) association) following its repeated presentation without consequences. Drug-naïve SZ patients during acute episodes (Baruch et al., 1988) and their first-degree relatives (Martins Serra et al., 2001) do not express LI (disregard the non-consequential pre-exposure of a stimulus and learn faster than controls to associate it with a significant US). Inability to ignore irrelevant stimuli (i.e., disrupted LI) is thought to associate with positive symptoms (Weiner, 2003; Morris et al., 2013), since drugs that disrupt LI (e.g., amphetamine) also exacerbate positive symptoms in SZ patients. A recent study (Knapman et al., 2010) revealed that LI deficits are also present in mice highly reactive to stress.

Here we evaluated LI expression in NrCAM-deficient mice (KO), an animal modeling genetic alterations associated with SZ, under no-stress (NS) and chronic unpredictable stress (CUS) conditions. We also compared neuronal activation (c-Fos+ cell counts) during the LI paradigm in brain regions previously shown to be relevant to LI (Schiller and Weiner, 2004; Gal et al., 2005; Schiller et al., 2006) in NrCAM KO mice and their wild-type (WT) littermates.

## 2 Materials and methods

### 2.1 Subjects

The subjects were forty 3–4 month-old male NrCAM-deficient (Sakurai et al., 2001) (KO, *n* = 20) mice and their *wild-type* littermate controls (WT, *n* = 20) obtained from breeding heterozygote NrCAM mice in a colony maintained on C57BL/6 J background for at least 10 generations. Genotypes were confirmed by PCR amplification from tail biopsy samples. The mice were further divided into *Stress* (S, *n* = 20) and *No-Stress* (NS, *n* = 20) groups. Mice were housed in a temperature-controlled room under a 12-h light–dark cycle. Mice were maintained at 85% of their *ad libitum* weights by restricting access to food (Purina 5001 Rodent Diet, Research Diets Inc., New Brunswick, NJ). All experimental procedures were conducted in accordance with the standards for the ethical treatment and approved by Utah State University IACUC Committee.

### 2.2 Chronic unpredictable stress

Stress mice received 21 days of CUS as in (Dias-Ferreira et al., 2009; Buhusi et al., 2017a), using the following daily randomly-chosen stressors applied at random daily times: 30 min restraint, 10 min forced swim, or 10 min exposure to an aggressive Balb/c male mouse. We have chosen this 3-week CUS protocol since stressed WT C57Bl/6 J mice seem to be resilient to this CUS (Buhusi et al., 2016, 2017a), and the aim was to comparatively evaluate NrCAM-deficient mice relative to their WT littermates. It should be noted that when exposed to a longer (8-week), more complex CUS protocol (Monteiro et al., 2015), C57Bl/6 J mice do show changes in anxiety, depressive-like, and exploratory behaviors.

### 2.3 Apparatus

The apparatus consisted of 8 standard mouse operant chambers housed inside sound-attenuating cubicles (Med Associates, St. Albans, VT) equipped with a house light, a fan, two nosepokes on the front wall and one nosepoke on the back wall, a programmable audio generator, a shocker/scrambler module, and a standard mouse 20-mg pellet feeder. The pre-exposed (PE) and non-pre-exposed (NPE) conditioned stimuli were a 80-dB tone and a 10-Hz click. The unconditioned stimulus was a 1-s 0.5 mA footshock.

### 2.4 Latent inhibition

Latent inhibition was assessed using an “on baseline” conditioned emotional response (CER) procedure consisting of baseline, pre-exposure, conditioning, rebaseline and test phases (i.e., allowing the mouse to eat during the all stages of the LI paradigm) (Buhusi et al., 2017a,b). Mice were assigned either to a PE tone / NPE click or PE click/NPE tone in a counterbalanced manner. Mice were shaped to nosepoke for food pellets on an FR1 schedule. The FR1 task was used as a “masking” task throughout the LI protocol, as often used in human LI studies (Braunstein-Bercovitz and Lubow, 1998; De la Casa and Lubow, 2001). The LI task consisted of four daily sessions as follows: During the 60-min pre-exposure session mice received forty 30-s presentations of the PE stimulus separated by a 60-s inter-stimulus interval (ISI). During the 30-min conditioning session, the PE and NPE stimuli were presented for 30 s three times, separated by a 240 s ISI. The last presentation of the PE and NPE stimuli was paired with a 1-s, 0.5-mA footshock. On the next day mice were given a 60-min rebaseline session during which mice were reinforced for nosepoking on an FR1 schedule. On the next day, during a 30-min test session, mice were presented with 3-min PE and NPE stimuli with an 8-min ISI (order counterbalanced between subjects). Mouse behavior was video recorded and the duration of freezing behavior was estimated using FreezeScan software (CleverSys Inc., Reston, VA) (Buhusi et al., 2017a,b, 2023).

### 2.5 c-Fos immunostaining

To evaluate neuronal activation, we performed c-Fos immunostaining using standard procedures (Buhusi et al., 2016, 2017a,b, 2023). Ninety min after the start of the test session mice were deeply anesthetized and transcardially perfused with a paraformaldehyde solution (4% in 0.1 M Phosphate buffer, pH 7.4). Brains were collected and sectioned on a vibrating microtome (VT1200S, Leica, Germany). Free-floating brain sections (50 μm) were permeabilized and incubated overnight at 4°C with the c-Fos primary antibody (Cell Signaling Technologies, 1:400 dilution). The next day sections were rinsed and incubated with Alexa488-conjugated goat anti-rabbit secondary antibody and NeuroTrace 530/615 (Fisher Scientific / Invitrogen, Carlsbad, CA). NeuroTrace neuronal labeling was used to identify the neuroanatomical regions of interest. Sections were rinsed in PBS before mounting with Prolong Diamond (Fisher Scientific/Invitrogen, Carlsbad, CA).

### 2.6 Neuronal activation analysis

Fluorescence images were acquired on a Zeiss LSM710 laser scanning confocal microscope using appropriate filter sets. Analysis of neuronal activation was performed by counting c-Fos-positive nuclei, in same-size areas in 1–2 sections/region of interest/mouse in the following areas of interest: prelimbic cortex (PrL: bregma 2.2–1.78), infralimbic cortex (IL: bregma 1.98–1.78), medial orbitofrontal cortex (mOFC: bregma 2.34–2.10), ventral orbitofrontal cortex (vOFC: bregma 2.34–2.10), nucleus accumbens shell (Acb-shell: bregma 1.33–1.09), and nucleus accumbens core (Acb-core: bregma 1.33–1.09) (Franklin and Paxinos, 2008), by independent observers unaware of genotype and LI performance. Neuronal activation in each region was subjected to statistical analyses.

### 2.7 Statistical analyses

The estimated duration of freezing behavior in the first 60 s of the presentation of the PE and NPE stimuli during the conditioning and test sessions was subjected to mixed ANOVAs with between-subjects variables stress (S, NS) and genotype (KO, WT), and within-subjects variable pre-exposure (PE, NPE), followed by *post-hoc* analyses. The latency to freeze (to the context) during the conditioning and test sessions was subjected to mixed ANOVAs with between-subjects variables stress (S, NS) and genotype (KO, WT), and within-subjects variable session (conditioning, test), followed by *post-hoc* analyses. The difference in freezing between NPE and PE, the number of rewards earned during the test session, and the neuronal activation (c-Fos+ cell counts) in each brain region were subjected to 2-way ANOVAs with factors stress (S, NS) and genotype (KO, WT), followed by LSD *post-hoc* analyses. All statistical analyses were conducted at an alpha level 0.05.

## 3 Results

### 3.1 Latent inhibition

Latent inhibition (LI) was assessed in NrCAM KO mice and WT littermates using a CER procedure consisting of baseline, pre-exposure, conditioning, rebaseline and test phases (Buhusi et al., 2017a,b). The CER procedure was cue-driven, since NrCAM KO mice display normal cue fear conditioning, although they show impairments in contextual fear (Matzel et al., 2008; Mohan et al., 2019a). The mice (KO and WT) were divided into no-stress (NS) and stress (S) groups; S mice received 21 days of CUS as in (Dias-Ferreira et al., 2009; Buhusi et al., 2017a) before being tested for LI.

**Figure 1:**
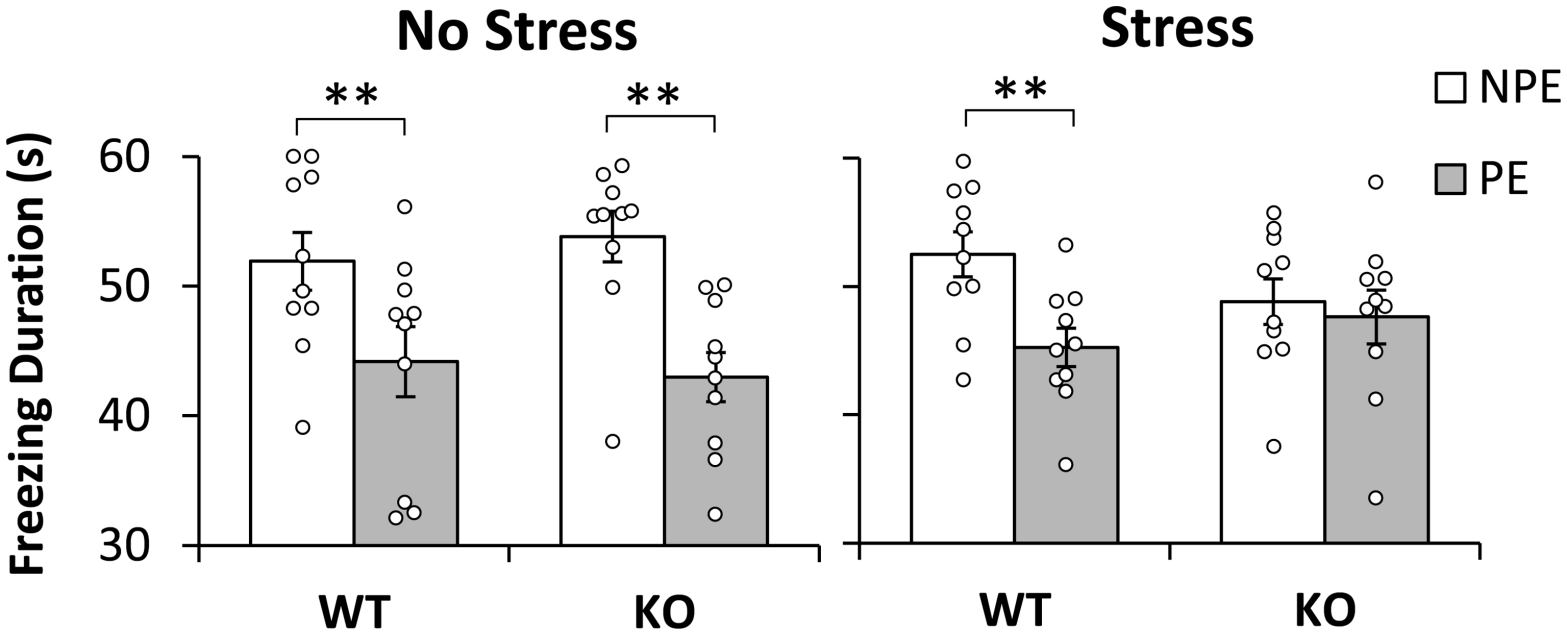
Latent inhibition by stress and genotype. Average duration of freezing (±SEM) to the pre-exposed (PE) and non-pre-exposed (NPE) stimuli in NrCAM knockout (KO) and wild type littermate controls (WT) under no-stress (left) and chronic unpredictable stress (right). A significant latent inhibition (significantly larger freezing to NPE than PE) was observed in all groups except in stressed NrCAM KO mice. ^*^p < 0.05; ^**^p < 0.01.

The average freezing duration during the first 60s of the presentation of the PE and NPE stimuli in the test session is shown in Figure 1. Analyses indicated a main effect of pre-exposure (*F*(1,36) = 37.239, *p* = 0.001), suggesting that mice froze longer during the NPE stimulus than during the PE stimulus (LI). However, LI was not expressed equally in all groups: Analyses indicated a significant pre-exposure x stress interaction (*F*(1,36) = 5.244, *p* = 0.028), suggesting that NS mice showed more LI—larger difference in freezing between NPE and PE stimuli—than S mice. Furthermore, analyses indicated a significant pre-exposure x stress x genotype interaction (*F*(1,36) = 4.280, *p* = 0.046), suggesting that stressed KO mice were particularly impaired in LI relative to the other groups. Planned comparisons indicated a significant difference in freezing between NPE and PE in No-Stress mice irrespective of genotype, NS-WT mice (*F*(1,36) = 12.223, *p* = 0.001) and NS-KO mice (*F*(1,36) = 23.974, *p* = 0.001). Planned comparisons also indicated a significant difference in freezing between NPE and PE in stressed S-WT mice (*F*(1,36) = 10.724, *p* = 0.002), but not in stressed S-NrCAM KO mice (*F*(1,36) < 1), indicating that all mice showed LI except stressed NrCAM KO mice. Taken together, these results provide support for a model under which environmental factors (stress) potentiate the effect of genotype to reveal the disruption of LI in stressed NrCAM KO mice but not in the other groups.

### 3.2 Unconditioned freezing

To evaluate the hypothesis that the difference in freezing to PE and NPE stimuli in Figure 1 may be due to the intrinsic (unconditioned) differences in freezing to the two stimuli, we performed analyses of freezing behavior to the PE and NPE stimuli in the conditioning session, before these stimuli were paired with footshock. These analyses failed to indicate any main effects of stimulus (PE/NPE) (*F*(1,36) < 1), or any interactions with the stimulus: stimulus x genotype (*F*(1,36) = 1.606, *p* = 0.213), stimulus x stress (*F*(1,36) = 2.344, *p* = 0.134), and stimulus x genotype x stress (*F*(1,36) = 3.163, *p* = 0.084), suggesting no differences in unconditioned freezing to the PE and NPE stimuli, irrespective of genotype and stress condition.

### 3.3 Reactivity to shock

Another possibility is that stressed NrCAM KO mice became more reactive to shock than the other groups. To evaluate this hypothesis we followed four lines of evidence: First, a *post-hoc* LSD test of the duration of freezing during the test session (see section 3.1) failed to indicate differences between genotypes in duration of freezing to the NPE stimulus (all *p*s > 0.201) (see Figure 1); same analyses also failed to indicate differences in duration of freezing to the NPE stimulus between unstressed and stressed mice for each genotype (all *p*s > 0.083) (see Figure 1).

Second, analyses of the latency to freeze in the conditioning session (before exposure to shock) and in the test session (after exposure to shock) failed to indicate any effects of session, genotype, stress, or any interactions (all *F*s(1,36) < 2.342, all *p*s > 0.135), suggesting that the propensity to freeze in the given context did not change after exposure to shock, and did not vary with stress and genotype, thus making it unlikely that mice differed in their reactivity to shock.

Third, analyses of the number of rewards earned during the pre-exposure session (before the shock), during conditioning, and during rebaseline and test sessions (after the shock) indicated an effect of session (*F*(3,108) = 456.304, *p* = 0.0001) suggesting that rewards differed by session. Planned comparisons indicated more rewards during both pre-exposure and rebaseline than during both conditioning and test sessions (*p*s < 0.0001). However, analyses failed to indicate any significant main effects or interactions with genotype and stress variables (*F*s(3,108) < 1.752, *p*s > 0.161), suggesting that mice earned food similarly irrespective of stress and genotype, thus making it unlikely that the absence of LI in stressed NrCAM KO mice is due to these mice being more reactive to shock than WT mice.

Fourth, analyses of the number of nosepokes during the pre-exposure session (before the shock), during conditioning, and during rebaseline and test sessions (after the shock) indicated an effect of session (*F*(3,108) = 29.981, *p* = 0.0001) suggesting that nosepoking differed by session. Planned comparisons indicated more nosepoking during both pre-exposure and rebaseline than during both conditioning and test sessions (*p*s < 0.0001). However, analyses failed to indicate any significant main effects or interactions with genotype and stress variables (*Fs*(3,108) < 1.664, *p*s > 0.198), suggesting that nosepoking was not affected by genotype or stress, thus making it unlikely that the absence of LI in stressed NrCAM KO mice is due to these mice being more reactive to shock than the WT mice.

### 3.4 Neuronal activation

**Figure 2:**
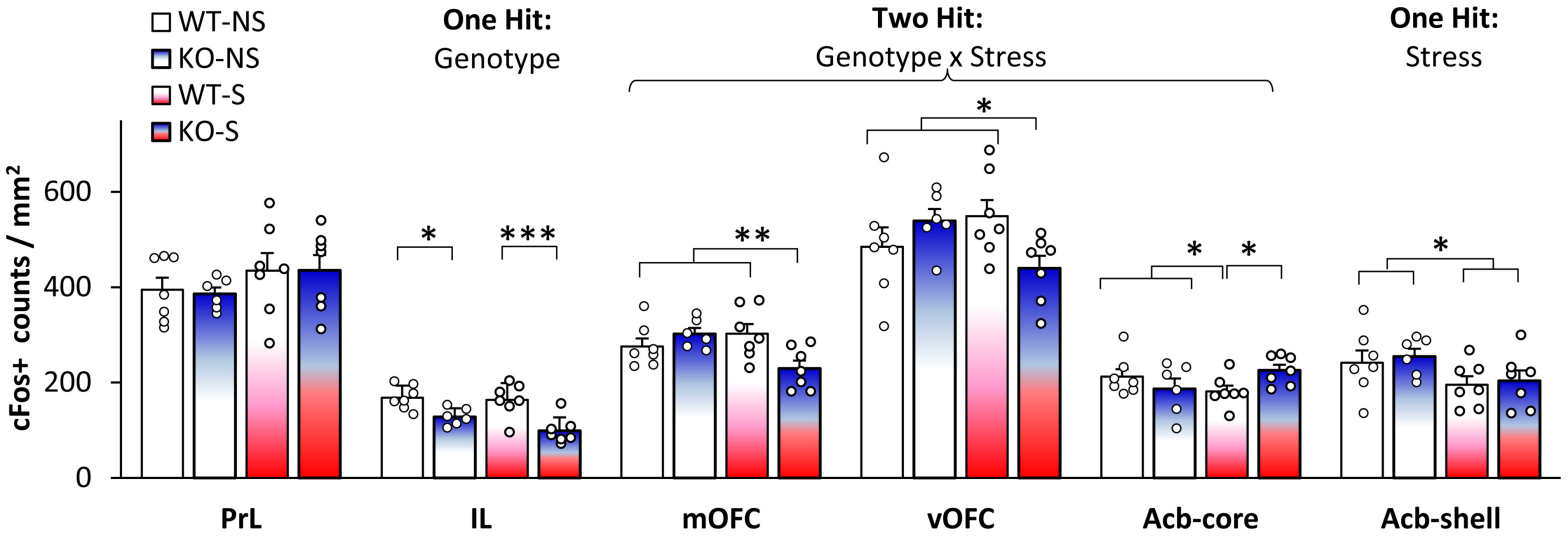
Neuronal activation during latent inhibition testing. Average c-Fos+ cell counts (±SEM) in prelimbic cortex (PrL), infralimbic cortex (IL), medial orbitofrontal cortex (mOFC), ventral orbitofrontal cortex (vOFC), nucleus accumbens core (Acb-core), and nucleus accumbens shell (Acb-shell) in no-stress (NS) and stress (S) NrCAM-deficient mice (KO) and wild-type littermate controls (WT). ^*^p < 0.05; ^**^p < 0.01; ^***^p < 0.001.

To evaluate which brain regions were differentially activated during LI (Buhusi et al., 2016, 2017a,b, 2023) we assessed neuronal activation through analyses of expression of the immediate early gene c-Fos. We focused on brain regions known to be relevant to LI through lesion or pharmacological studies (Yee et al., 1995; Pouzet et al., 2004; Schiller and Weiner, 2004; Gal et al., 2005; Schiller et al., 2006; Ouhaz et al., 2014). Figure 2 indicates four types of effects of stress and genotype on neuronal activation in these brain regions: a single hit by genotype in IL cortex, a single hit by stress in Acb-shell, a dual hit stress x genotype interaction in mOFC, vOFC, and Acb-core, and no effect in PrL cortex. First, analyses indicated a main effect of genotype in IL cortex (*F*(1,23) = 24.267, *p* = 0.0001), but no other main effects or interactions (*F*s(1,23) < 2.495, *p*s > 0.128). Second, analyses indicated a main effect of stress in Acb-shell (*F*(1,23) = 5.307, *p* = 0.031), but no other main effects or interactions (*F*s(1,23) < 1). Third, analyses indicated stress x genotype interactions in mOFC (*F*(1,23) = 8.428, *p* = 0.008), vOFC (*F*(1,23) = 6.245, *p* = 0.020), and Acb-core (*F*(1,23) = 5.199, *p* = 0.032), but no other main effects (*F*s(1,23) < 1.852, *p*s > 0.187). LSD *post-hoc* analyses failed to indicate differences in neuronal activation between KOs and WTs in the no-stress condition in either mOFC (*p* = 0.296), vOFC (*p* = 0.259), or Acb-core (*p* = 0.255); differences between KOs and WTs emerged only under stress: mOFC (*p* = 0.005), vOFC (*p* = 0.024), or Acb-core (*p* = 0.049), indicating that NrCAM KO mice are vulnerable to stress (neuronal activation in NrCAM KO mice becomes different from WT’s only under stress). Finally, analyses of c-Fos counts in PrL cortex failed to indicate any main effects or interactions (*Fs*(1,23) < 2.325, *p* > 0.141). These results indicating a pattern of differential effects of genotype and stress support a complex stress x genotype interaction model.

## 4 Discussion

Using an “on baseline” within-subject CER LI procedure developed in our lab (Buhusi et al., 2017a,b, 2023), the current study found that C57BL/6 J WT mice showed LI, irrespective of stress, consistent with previous findings (Gould and Wehner, 1999; Buhusi et al., 2017a). Additionally, results indicated that NrCAM KO mice showed LI under baseline, no-stress conditions, but not after exposure to a CUS regimen. These results were not due to differences in unconditioned freezing to the two stimuli, thus describing true differences in LI. Moreover, these results were not due to differences in reactivity to shock, as all mice froze similarly to the two stimuli (before they were paired with shock), nosepoked similarly with the other mice both before and after being exposed to shock, learned similarly about the NPE stimulus and context irrespective of exposure to shock, and were rewarded similarly during the task. Further studies are required to evaluate whether altered LI as a consequence of the stress x NrCAM-deficit interaction reflects anomalies in either acquisition (stimulus pre-exposure) or expression of LI.

Neuronal activation analyses (c-Fos+ cell counts) in stressed mice in brain regions involved in LI indicated that in some brain regions activity decreased in KO mice relative to WTs (IL, mOFC, and vOFC), in other regions activity increased (Acb-core), while in others it was not affected by genotype (PrL and Acb-shell). The absence of differences in the PrL and Acb-shell activation between genotypes in the LI task further suggests that in our study the changes in neuronal activity were not general, but were rather specific to certain brain areas. Our results reveal that stress and genetic factors interact to alter neuronal activation in mOFC, vOFC, and the Acb-core, and support current neurobiological (Weiner, 2003; Lingawi et al., 2017) and neuro-computational models of LI (Schmajuk et al., 1997; Buhusi et al., 1998; Schmajuk et al., 1998).

### 4.1 Neural substrates of latent inhibition

Acb is a key structure in LI acquisition and expression. Lesion studies support opposing roles of Acb-shell and core in LI: lesions of the Acb-shell impair LI (Weiner et al., 1999), while lesions of Acb-core or Acb-shell+core are associated with persistent LI (expression of LI under conditions where normal animals do not exhibit LI) (Weiner et al., 1999; Gal et al., 2005). Results of neuronal activation presented in Figure 2 provide evidence for an effect of stress in the Acb-shell, and for a two hit, stress x genotype interaction, in the Acb-core, and suggest an increased vulnerability of NrCAM mice to the effect of stress in LI.

The involvement of the prefrontal cortex, which is bi-directionally connected with the hippocampus and amygdala and projects to the Acb (Del Arco and Mora, 2008, 2009), has also been evaluated in relation to LI, with mixed results: Excitotoxic lesions of the medial prefrontal cortex do not affect LI (Lacroix et al., 1998). However, OFC lesions, a region involved in behavioral flexibility (Rudebeck et al., 2013), lead to persistent LI (Schiller and Weiner, 2004), while temporary chemogenetic OFC inactivation during pre-exposure disrupts LI (Costa et al., 2021). Here, we observed a stress x genotype interaction in OFC activation, with reduced OFC activation in stressed NrCAM KO showing disrupted LI. Our results may be explained either by reduced discrimination between cues (Sarlitto et al., 2018) or by poor inference of outcomes (Rudebeck and Murray, 2014; Boorman et al., 2021). For example, in the present study, the disruption in LI in NrCAM KO relative to WTs may have been mediated by (opposing) changes in freezing to the PE and NPE stimuli.

**Figure 3:**
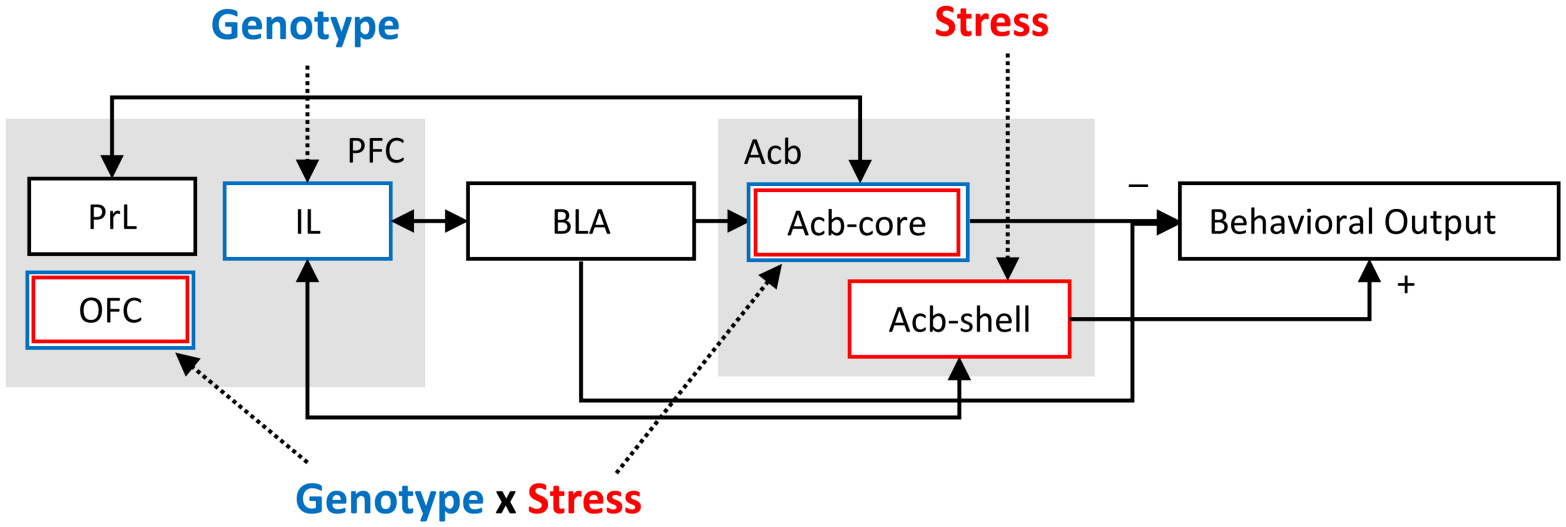
Model of the impact of NrCAM and stress on a latent inhibition circuit (modified from Schmajuk et al., 1997; Weiner and Arad, 2009). IL cortex was found to be vulnerable to the NrCAM genotype irrespective of stress. Acb-shell was found to be vulnerable to the effect of stress irrespective of genotype. mOFC, vOFC, and ACb-core were found to be vulnerable to stress only in NrCAM KO mice (two hit, genotype x stress interaction). Instead, PrL cortex was not found to be affected by either genotype or stress in the present LI paradigm. PFC, prefrontal cortex; PrL, prelimbic cortex; IL, infralimbic cortex; OFC, orbitofrontal cortex; BLA, basolateral amygdala; Acb, nucleus accumbens; Acb-core, nucleus accumbens core; Acb-shell, nucleus accumbens shell.

The infralimbic cortex (IL) is an important prefrontal cortex region for fear regulation (Izquierdo et al., 2016). It was proposed that during LI testing, the initial inhibitory memory established by pre-exposure is reactivated in the IL; this memory is strengthened by pharmacological IL stimulation, and disrupted by NMDA receptor blockade (Lingawi et al., 2017). Interestingly, in our study, IL neuronal activation is significantly decreased in NrCAM KO mice relative to WT irrespective of stress, supporting the above hypothesis and possibly reflecting alterations in excitation-inhibition balance in IL cortex. Figure 3 shows a diagrammatic model summarizing the role of NrCAM genotype, stress, and their interaction on a latent inhibition circuit (modified from Schmajuk et al., 1997; Weiner and Arad, 2009). Figure 3 indicates that IL cortex is altered by the NrCAM genotype irrespective of stress, that Acb-shell is vulnerable to the effect of stress irrespective of genotype, and that OFC and ACb-core are vulnerable to the stress only in NrCAM KO mice (two hit genotype x stress interaction). Instead, PrL cortex does not show vulnerabilities to either genotype or stress in our LI paradigm.

### 4.2 Stress, latent inhibition and schizophrenia

Stress initiates complex organismal responses to ensure adaptation and survival of the individual. Acute stress usually induces adaptive time-limited responses, while persistent changes resulting from long-term chronic stress have deleterious implications for health (de Kloet et al., 2005; Pardon and Marsden, 2008; Herman, 2013) and cognition, in both humans (Lupien et al., 2007; Marin et al., 2011) and experimental animals (Holmes and Wellman, 2009; Moreira et al., 2016). Stress attenuates LI in humans (Braunstein-Bercovitz et al., 2001), rats (Hellman et al., 1983), and mice highly reactive to stress (Knapman et al., 2010). In vulnerable individuals, chronic stress can precipitate psychiatric disorders (Bale, 2006; Deppermann et al., 2014; Nestler et al., 2016), including schizophrenia (Aiello et al., 2012; Holtzman et al., 2012, 2013).

Rodent models of chronic stress exhibit alterations of dendrite morphology, including reductions in dendrite complexity and spine density in the hippocampus and prefrontal cortex but increases in the basolateral amygdala and nucleus accumbens. Alterations of spine density and synapse connectivity in these regions may contribute to disruption of cognition, emotion, motivation, and reward in animal models and humans (Duman and Duman, 2015).

Genetic and epigenetic factors are major contributors to vulnerability or resilience to stress (Ising and Holsboer, 2006; Cahill et al., 2022). For example, recent studies identified a major role for the glial-derived neurotrophic factor (GDNF) in response to chronic unpredictable stress (CUS): Increased GDNF expression in nucleus accumbens and hippocampus promotes resilience and recovery from CUS (Uchida et al., 2010). Instead, animal models which cannot up-regulate GDNF during stress exhibit anxiety, anhedonia (Bian et al., 2012) and disrupted LI (Buhusi et al., 2017a). In our current study only stressed NrCAM KO mice, but not stressed wild-type littermates, failed to express LI.

### 4.3 NrCAM and schizophrenia

NrCAM is a cell adhesion molecule, widely expressed in the nervous system, and with important physiological functions during neurodevelopment (Grumet et al., 1991; Grumet, 1997; Sakurai et al., 2001; Sakurai, 2012), from axon guidance and circuit formation (Falk et al., 2005; Williams et al., 2006; Dai et al., 2013), to spine pruning (Mohan et al., 2019b; Duncan et al., 2021) or synapse stabilization (Demyanenko et al., 2014; Mohan et al., 2018; Takano et al., 2020), to organization of the nodes of Ranvier (Custer et al., 2003; Feinberg et al., 2010). NrCAM lies at the core of a functional protein network where multiple targets have been associated with schizophrenia (Ayalew et al., 2012; Stevens and Rasband, 2021).

Beside its major neurodevelopmental roles, NrCAM was recently found to be one of the genes regulated by stress in rodents (Olsen et al., 2022); the same study reports that a genetic variant of the human NrCAM gene is associated with negative affect as a result of abusive supervision (Olsen et al., 2022). Interestingly, NrCAM is involved in spine pruning (Mohan et al., 2019b; Duncan et al., 2021) and synapse remodeling, a phenomenon reported in rodent models of chronic stress (Duman and Duman, 2015; McEwen et al., 2016); it is thus plausible that NrCAM alterations are related to the exaggerated synaptic pruning seen during adolescence and young adulthood in SZ (Selemon and Zecevic, 2015; Sellgren et al., 2019; Howes and Onwordi, 2023). Together, these findings support a role for NrCAM in neurodevelopment and vulnerability to environmental stressors, and support the significance of our current study, linking NrCAM to a cognitive endophenotype relevant to SZ.

### 4.4 Limitations and extensions

Here are some limitations, or departures of this study from the literature. First, while traditional CER LI paradigms measure the effect of pre-exposure on a behavioral response (e.g., suppression of lever pressing etc.), in the current study nosepoking in FR1 task was only used as a “masking” task. Instead, the current study directly measured freezing from video recordings using a computer program. To better align our protocol with traditional CER protocols, future studies could measure both the direct effect of pre-exposure on freezing (measured from video recordings) as well as its indirect effect on nosepoking, and estimate whether these two measures correlate. Second, this investigation was conducted in homozygote NrCAM KO mice as an animal model of SZ, rather than in SZ patients, which are more likely to be heterozygotes for NrCAM gene alterations. As such, future studies could also investigate the effect of stress on LI in NrCAM heterozygote mice, which may align our protocol with future human studies.

On the other hand, the results of the present study could be extended to other disorders, since LI is also impaired in ADHD (Lubow et al., 2014), Parkinson’s disease (Gyorfi et al., 2017), depression (Lazar et al., 2012), and OCD (Kaplan et al., 2006). As such, the current study could provide a future avenue to study the effect of stress on the impairments in selective attention in these conditions. Finally, the current study may also provide clues regarding the roles of other cell adhesion molecules (e.g., CHL1, Buhusi et al., 2017b), or other molecules involved in neurodevelopment (e.g., GDNF, Buhusi et al., 2017a; or BDNF, Buhusi et al., 2023) in impairments of selective attention related to neurodevelopmental disorders.

## 5 Conclusion

This study identified a disruption of LI in NrCAM-deficient mice after chronic unpredictable stress, associated with reduced neuronal activation in IL and OFC, and increased neuronal activation in Acb-core, linking NrCAM to a cognitive endophenotype relevant to SZ. The disruption of LI may be the result of genotype-related alterations in the balance of excitation-inhibition in cortical circuits or in neuronal connectivity, both of which may be potentiated as a result of chronic stress. NrCAM has been documented as influencing vulnerability to stress, for example regarding stress-induced headaches (Sannes et al., 2023), stress-induced changes in pituitary function (Olsen et al., 2022), and stress-induced changes in hippocampal neurogenesis (Li et al., 2021). The current study adds to this list that NrCAM is linked to a vulnerability to chronic unpredictable stress associated with impaired latent inhibition, a phenotype relevant to acute schizophrenia-like symptoms.

## Data availability statement

The raw data supporting the conclusions of this article will be made available by the authors, without undue reservation.

## Ethics statement

The animal study was approved by Utah State University IACUC Committee. The study was conducted in accordance with the local legislation and institutional requirements.

## Author contributions

MB: Conceptualization, Formal analysis, Funding acquisition, Investigation, Methodology, Project administration, Resources, Supervision, Validation, Visualization, Writing – original draft, Writing – review & editing. CKB: Investigation, Writing – review & editing. CVB: Conceptualization, Data curation, Formal analysis, Funding acquisition, Investigation, Methodology, Project administration, Resources, Software, Supervision, Validation, Visualization, Writing – original draft, Writing – review & editing.
